# The relationship between parental phubbing and mobile phone addiction in junior high school students: A moderated mediation model

**DOI:** 10.3389/fpsyg.2023.1117221

**Published:** 2023-04-12

**Authors:** Zhenhong Mi, Wanjun Cao, Wenjing Diao, Meixiu Wu, Xin Fang

**Affiliations:** ^1^Student Counselling and Mental Health Center, Qingdao University, Qingdao, China; ^2^Department of Psychology, Normal College of Qingdao University, Qingdao, China

**Keywords:** parental phubbing, mobile phone addiction, parent-child cohesion, friendship quality, junior high school students

## 1. Introduction

The well-developed information technology has fully integrated into people's everyday lives. By June 2021, the number of young Internet users between the ages of 10 and 19 accounted for 12.3% of the total in China. Although the mobile phone brings benefits, it has repercussions especially for adolescents, for instance, mobile phone addiction. An increasing number of adolescents cannot live without mobile phones and are addicted to them.

In addition, due to their immature thinking, low cognitive abilities and lack of social experience (Shan, 2018), junior high school students are susceptible to mobile phone addiction. Moreover, mobile phone addiction also has some association on their emotional development, studies, interpersonal communication, parent-child relationships, and even personal safety (Višnjić et al., 2018). Specifically, young people's higher score of mobile phone addiction is positively associated with their surroundings, such as family atmosphere, negative parenting practices (Ma et al., 2021; Ding et al., 2022; Jiang et al., 2022). Therefore, it is significant to explore and test the factors that influence mobile phone addiction.

Previous studies have found that environmental factors (e.g., family, social support, and peer interactions) have an impact on mobile phone addiction (Ge and Zhu, 2014; He et al., 2020; Yang et al., 2022). Although previous studies have been copious, some studies have examined mobile phone addiction in relation to parental phubbing, a particularly negative parenting practices with an insidious way (Xie and Xie, 2020; Zhang et al., 2021).

Karadag and Sule Betül Tosunta (2015) proposed that phubbing refers to avoiding interpersonal communication by not paying attention to each other when they are dealing with others, and avoiding interpersonal communication by looking down at the phone to deal with related matters. Most scholars associate phubbing with individuals who have no time to pay attention to others because of the use of mobile phones, and it may affect the quality of interpersonal communication. Roberts and David (2016) argue that the situation where the social process stops halfway through playing with a mobile phone can be called phubbing. Other scholars believe that phubbing refers to the behavior of peers being ignored and snubbed because the other party focuses on the mobile phone (Chotpitayasunondh and Douglas, 2018). Although scholars have different expressions of phubbing, they all emphasize the neglect of others in interpersonal communication.

Parental phubbing indicates a new phenomenon that taking place during parent-child interactions, was defined as that parents snub or neglect their children in social settings by concentrating on phone use (Xie et al., 2019). Based on the above explanation, parental phubbing was considered a negative parenting practice (e.g., harsh parenting, rejection, parenting by lying and neglect). Among the many influencing factors, parenting practice has attracted the attention of many researchers. They found that negative parenting practice increases the risk of mobile phone addiction (Sun et al., 2019; Zhang et al., 2021; Wei et al., 2022a,b).

### 1.1. Parental phubbing and mobile phone addiction

Parental acceptance-rejection theory (PAR Theory), proposed and developed by Rohner (1977), is a systematic attempt to understand how early experiences of parental acceptance-rejection affect emotional, behavioral, and social-cognitive development of children (Rohner, 1998). One of the questions Rohner addresses is whether the effects of perceived parental acceptance-rejection extend from childhood through adolescence and into adulthood. Children who perceive them to be rejected by attachment figures—particularly their parents -are likely to develop some psychological problems (Rohner, 2004; Rohner et al., 2005). The emotional need for the positive response from significant others is a powerful motivator, and when children do not get this need satisfied adequately by their parents, they are likely to respond emotionally and behaviorally in specific ways (Rohner, 2004). When children do not feel enough emotional attachment from their parents, they may seek it elsewhere. Nowadays, adolescents often turn to the mobile phone for the feelings of being loved and respected (Wei et al., 2022b). Some parents exhibit “enthusiasm” for their mobile phones and thus “neglect” their children. This behavior not only sets a bad “example” for junior high school students but also results in emotional “neglect,” which can increase junior high school students' addiction to mobile phones. Given that the current Chinese adolescents access the mobile phones more easily, we assume that adolescents who have experienced neglected by parental phubbing are likely to develop mobile phone addiction due to over-reliance on mobile phones. Thereby, based on the theoretical and empirical evidence stated above, we propose H1.

Hypothesis 1: Parental phubbing was positively associated with adolescents' mobile phone addiction.

### 1.2. The mediation of parent-child cohesion

Firstly, parental phubbing may affect parent-child cohesion. Parent-child cohesion, an essential indicator of the quality of parent-child relationships, is generally defined as the intimate emotional bonding between children and their parents (Wang et al., 2022). Parent-child cohesion reflects the degree of supportive interactions within the parent-child system, such as talking about worries, joining discussions, and so on (Wang et al., 2022). The “Substitution Hypothesis” (Coyne et al., 2011) also claims that the time during which parents bow their heads and play with their mobile phones may replace and reduce the time that they should have spent interacting with their children, and that such families may have a relatively lower degree of parent-child cohesion than those families in which there are fewer parental phubbing.

Secondly, parent-child cohesion may affect mobile phone addiction. The family plays a central role in the socialization process of adolescents, and parents are expected to provide an emotional bond and behavioral constraints (Lau et al., 1990; Gray and Steinberg, 1999). Parental phubbing is associated with poor parent-child relationship and reduces the degree of parent-child cohesion (Xie et al., 2019; Niu et al., 2020). In poor parent-child cohesion family, children may feel a loss of their parents' love, warmth and other emotions supports (Wei et al., 2022b). As such, unsatisfied psychological needs will exacerbate the risk of problematic Internet use. According to the parental acceptance-rejection theory (Rohner, 2004), if children's needs are not met and they feel neglected in a family with negatively parent-child relationship, they may seek psychological satisfaction in other ways, such as the Internet, unusual social interactions, and mobile phones addiction. Therefore, we have assumed that satisfactory parent-child cohesion would be negatively related to adolescent mobile phone addiction.

Previous studies have directly demonstrated that in every way, children need a specific form of positive response—acceptance—from caregivers, especially parents (Chen et al., 2000; Khaleque and Rohner, 2002; Veneziano, 2003). In family life, parental phubbing is one of the disadvantages of the parent-child relationship and has a terrible effect on the family environment (Niu et al., 2020; Wang et al., 2020). They are too engrossed in their mobile phones instead of investing time and energy in their children to accompany them as they grow, which weakens parent-child cohesion (Geng et al., 2021). If children feel rejected rather than accepted by their parents, the parent-child relationship will become increasingly strained and the child will have to find other ways to get the attention and affection they are not getting from their parents, such as becoming addicted to mobile phones. Thereby, we proposed H2.

Hypothesis 2: Parent-child cohesion plays a mediating role in the relationship between parental phubbing and adolescents' mobile phone addiction.

### 1.3. The moderation of friendship quality

According to the model of individual ↔ context relations, we consider that friendship quality may moderate the association between parent-child cohesion and mobile phone addiction. The model of individual ↔ context relations hold that individuals' behaviors are formulated and developed in their interaction with their environment (Lerner et al., 2006). As individuals transition from childhood to adolescence, the role of peers becomes increasingly prominent in the process of socialization (Gaertner et al., 2010). Friendship quality, an essential indicator of the quality of friend relationships, reflects the degree of support provided among peers (Gauze et al., 1996). Previous studies have demonstrated that high-quality friendships provide important social support to adolescents and help reduce the occurrence of internalization and externalization problems in adolescents (Ostrov and Kamper, 2015; Fan et al., 2018). As an important support resource, high-quality friendships can provide youth with peer care and peer support (Troop-Gordon et al., 2015).

According to the model of individual ↔ context relations (Lerner et al., 2006), individuals' behaviors are formulated and developed in their interaction with their environment, when adolescents have difficulty establishing close relationships with peers, it can cause him to satisfy psychological needs such as belonging and support through undesirable peers and get caught up in undesirable peer interactions (Song et al., 2017), and poor peer interactions tend to exacerbate individual misbehavior problems (Xie et al., 2019). Conversely, previous studies have found that with a high friendship quality, adolescents will reduce anxiety levels due to parental neglect (Zhu et al., 2020). Good peer relationships can compensate for the negative effects of poor parent-child relationships (Tian and Tian, 2014). That is, a good friendship relationship can compensate for the harm caused to children by an unsatisfied parent-child relationship to a certain extent. Compared to adolescents with low friendship quality, adolescents with high friendship quality are able to have more opportunities to express themselves and feel peer support in their interactions with their friends (Ladd et al., 1996). This opportunity can reduce adolescent behavioral problems (Tian et al., 2018), and may reduce mobile phone addiction.

Based on the above research, we found that both parent-child cohesion and friendship quality were negatively related with mobile phone addiction. Satisfactory parent-child relationships predict less mobile phone addiction, and when adolescents not only have good parent-child relationships but also have high quality friendships, they receive not only parental care but also more social support, thus reducing mobile phone addiction. Therefore, friendship quality may play a moderating role between parent-child cohesion and mobile phone addiction. Compared with individuals with low friendship quality, the negative effect of parent-child cohesion on mobile phone addiction is stronger in individuals with high friendship quality. There by, we proposed hypothesis H3.

Hypothesis H3: Friendship quality moderates the relationship between parent-child cohesion and adolescents' mobile phone addiction.

### 1.4. The present study

The purpose of this study was 3-fold. First, we examined whether parental phubbing predicts adolescents' mobile phone addiction by proposing Hypothesis 1.

Hypothesis 1: Parental phubbing was positively associated with adolescents' mobile phone addiction.

Subsequently, we tested a mediator model to examine how parental phubbing is correlated to adolescents' mobile phone addiction by proposing Hypothesis 2.

Hypothesis 2: Parent-child cohesion plays a mediating role in the relationship between parental phubbing and adolescents' mobile phone addiction.

Finally, we examined whether subject moderated the relationship parent-child cohesion between and adolescents' mobile phone addiction, by advancing Hypothesis 3.

Hypothesis 3: Friendship quality moderates the relationship between parent-child cohesion and adolescents' mobile phone addiction.

**Figure 1:**
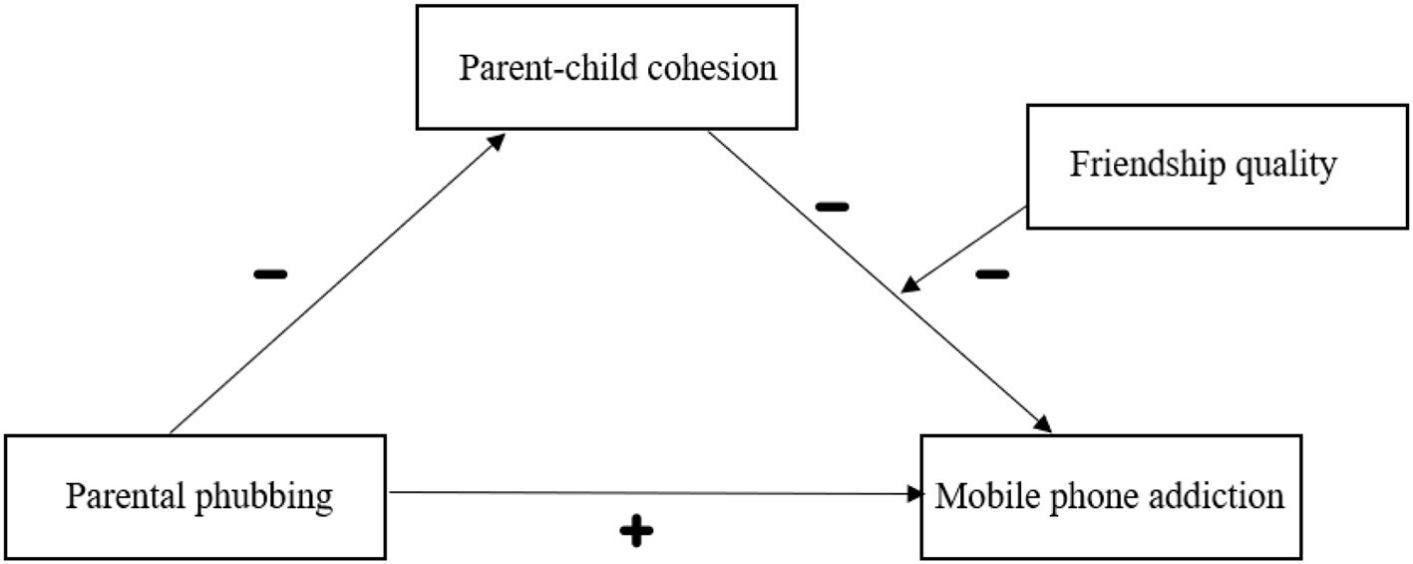
The hypothetical model.

Please see the hypothetical model in Figure 1.

## 2. Materials and methods

### 2.1. Participants

We recruited participants in each grade from grade 7 through grade 9 at random in a junior high school in China. After excluding unqualified samples, we finally collected 780 valid questionnaires with an effective response rate of 93.98% from 830 primary questionnaires. Participants included 339 students in grade 7 (*M*_*age of Grade* 7_ = 13.21, *SD*_*age of Grade* 7_= 0.52), 261 students in grade 7 (*M*_*age of Grade* 8_=14.38, *SD*_*age of Grade* 8_ = 0.54) and 180 students in grade 9 (*M*_*age of Grade* 9_=15.13, *SD*_*age of Grade* 9_ =0.35). We adopted convenience sampling in the current study. Of the participants, 393 (50.38%) were boys and 387 (49.62%) were girls (*M*_age_ = 14.04, *SD*_age_ = 0.93). Table 1 displays the demographic characteristics of the participants.

**Table 1:** Demographic characteristics of the participants.

	**No**.	**Percentage**
**Gender**		
Male	393	50.4%
Female	387	49.6%
**Grade**		
7	339	43.5%
8	261	33.5%
9	180	23%
**Sibling status**		
Only child	474	60.8%
Having one or more siblings	306	39.2%

### 2.2. Procedure

Consent was obtained from participants and their parents for the use of their responses in our research. The study was approved by the Ethical Committee for Scientific Research of the institution to which the authors are affiliated and was conducted by trained graduate students. Prior to formal data collection, these graduate students were trained to be familiar with the whole process of this survey, such as describing the instructions, explaining obscure items to some participants, and explaining the voluntary nature and confidentiality of participation. Participants were also asked to check the completeness of their responses on completion of the questionnaire. We measured students' demographic characteristics, parental phubbing, mobile phone addiction, parent-child cohesion, and friendship quality.

### 2.3. Measures

#### 2.3.1. Parental phubbing

Parental phubbing was assessed by the Parental Phubbing Scale (Roberts and David, 2016; Chinese version: Ding et al., 2020). It consists of 9 items rated on a five-point scale [e.g., “During a typical mealtime that my parents and I spend together, they pull out and checks their cell phone (slight modification)”]. The higher the total score is, the more serious the “bow-down” phenomenon is. In this study, the Cronbach's α of the total scale was 0.89.

#### 2.3.2. Mobile phone addiction

We measured mobile phone addiction with the 16-item Mobile Phone Addiction Scale (Xiong et al., 2012). A 5-point Likert scale was adopted (e.g., “Rather than communicate directly face to face, I prefer to chat on my cell phone”). The higher the total score is, the higher the students' level of addiction to mobile phones is. In this study, the Cronbach's α of the total scale was 0.90.

#### 2.3.3. Parent-child cohesion

Parent-child Scale was compiled by Olson et al. (1979) and revised by Zhang et al. (2006). It includes 10 items with five-point rating. This scale includes two subscales, father and mother, which features the same content [e.g., “Father (mother) and I have common interests and hobbies”]. The scores of father-child cohesion and mother-child cohesion are calculated, and the average scores of the two scales are taken as the total score of parent-child cohesion. The higher this score is, the better the parent-child relationship is. In this study, the Cronbach's α of the total scale was 0.94.

#### 2.3.4. Friendship quality

We assessed friendship quality with the 38-item Friendship Quality Questionnaire (Parker and Asher, 1993; Chinese version: Zou et al., 1998). The scale was divided into five dimensions, namely, trust and support, companionship and entertainment, affirmation of value, intimate exposure and communication, conflict and betrayal (e.g., “Between classes we always chat or play together”). The average of all questions is used to represent the friendship quality of children. The higher the score is, the better the friendship quality of the subjects is. In this study, the Cronbach's α of the total scale was 0.93.

## 3. Statistical analyses

First, we employed Harman single-factor test to conduct whether common method bias exists in this study. The results of principal component factor analysis without rotation showed that there were 13 factors whose eigenvalues were greater than 1. The variance explained by the first factor was 18.75%, below the threshold of 40% (Podsakoff et al., 2003). Therefore, the common method bias did not affect the outcome of this study.

Second, descriptive analysis was used to examine the study variables, and Pearson correlation analysis was used to examine the correlations between variables.

Third, PROCESS version 3.3 (Hayes, 2018) was used to test the moderated mediating model. Model 82 was used in PROCESS to test the mediating role of parent-child cohesion (mediator) and the moderated role of friendship quality (moderator) in the relationship between parental phubbing (independent variable) and mobile phone addiction (dependent variable). We used 5,000 bootstrap samples and the 95% bias-corrected confidence interval (95% CI) to examine the significance of the moderated mediating effect (Hayes, 2018). The statistical significance level was set at *p* < 0.05. Gender and age were also controlled for in the analysis.

## 4. Results

### 4.1. Preliminary analysis

Table 2 presents the Pearson correlations, means, and standard deviations of all variables. As Table 2 indicates, parental phubbing was positively correlated with mobile phone addiction. Parent-child cohesion was negatively correlated with parental phubbing and mobile phone addiction.

**Table 2:** Descriptive statistics and correlations between main variables.

**Variables**	***M***	***SD***	**1**	**2**	**3**	**4**
1 Parental phubbing	7.66	0.90	–			
2 Parent-child cohesion	3.10	0.82	−0.49^**^	–		
3 Mobile phone addiction	1.87	0.76	0.54^**^	−0.53^**^	-	
4 Friendship quality	3.42	0.70	0.01	0.25^**^	−0.15^**^	-

N = 780;

** p < 0.01.

### 4.2. Mediation analysis

Model 4 of PROCESS (Hayes, 2013) was used to examine the possible association between parental phubbing and mobile phone addiction as well as the possible mediating effect of parent-child cohesion. The results of the mediation analysis are presented in Table 3. After controlling for age and gender, we first found that parental phubbing positively predicted mobile phone addiction, *B* = 0.53, *p* < 0.001 (Eq. 1). Second, parental phubbing negatively predicted parent-child cohesion, *B* = −0.54, *p* < 0.001 (Eq. 2). Third, parental phubbing positively predicted mobile phone addiction, *B* = 0.32, *p* < 0.001, parent-child cohesion negatively predicted mobile phone addiction, *B* = −0.38, *p* < 0.001 (Eq. 3). Finally, the bias-corrected bootstrapping mediation test indicated that the process by which parental phubbing predicted mobile phone addiction through parent-child cohesion was significant, indirect effect = 0.17, 95% CI = [0.14, 0.21], which are presented in Table 4.

**Table 3:** The mediation model.

**Predictors**	**Equation 1 (criterion** = **mobile phone addiction)**		**Equation 2 (criterion** = **parent-child cohesion)**		**Equation 3 (criterion** = **mobile phone addiction)**	
	***B***	***t***	***B***	***t***	***B***	***t***
Parental phubbing	0.53	17.26^***^	−0.54	−17.62^***^	0.32	9.68^***^
Parent–child cohesion					−0.38	−11.33^***^
Age	−0.10	−3.16^**^	−0.18	−5.91^***^	−0.17	−5.69^***^
Gender	0.07	2.36*	−0.20	−6.56^***^	−0.00	−0.12
*R^2^*	0.31		0.30		0.40	
*F*	114.09^***^		111.39^***^		131.69^***^	

N = 780,

* p < 0.05;

** p < 0.01;

*** p < 0.001.

**Table 4:** Bia-corrected bootstrapping test in mediating effect.

**Pathways**	**Effect**	**95% confidence interval**		**Percentage**
		**Boot LLCI**	**Boot ULCI**	
Direct path				
Parental phubbing → mobile phone addiction	0.28	0.22	0.33	62%
Indirect path				
Parental phubbing → parent-child cohesion → mobile phone addiction	0.17	0.14	0.21	38%

The results of the mediation analysis support H1 and H2.

### 4.3. Moderation analysis

**Figure 2:**
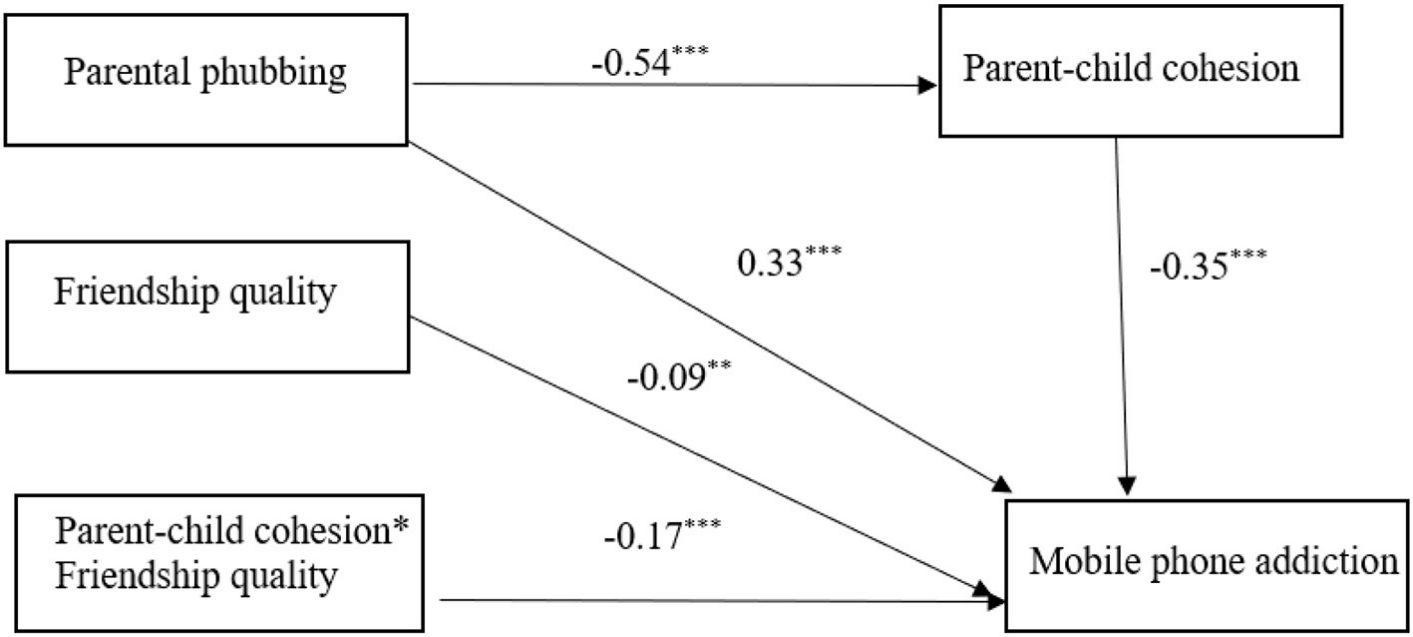
The interaction of parent-child cohesion and friendship quality on mobile phone addiction. **p < 0.01; ***p < 0.001.

We used Model 14 of PROCESS (Hayes, 2013) to examine whether friendship quality moderated the association between parent-child cohesion and mobile phone addiction. The results of the moderation analysis are presented in Table 5 and Figure 2. The regression model indicated that the interaction between parent-child cohesion and friendship quality was negatively associated with mobile phone addiction (*B* = −0.17, *p* < 0.001).

**Table 5:** The moderation model.

**Predictors**	**Equation 1 (criterion** = **mobile phone addiction)**	
	***B***	***t***
Parental phubbing	0.33	10.07^***^
Parent–child cohesion	−0.35	−10.48^***^
Friendship quality	−0.09	−3.07^**^
Age	−0.16	−5.50^***^
Gender	−0.05	−0.93
Parent–child cohesion* friendship quality	−0.17	−6.21^***^
*R^2^*	0.44	
*F*	100.62^***^	

N = 780,

** p < 0.01;

*** p < 0.001.

**Figure 3:**
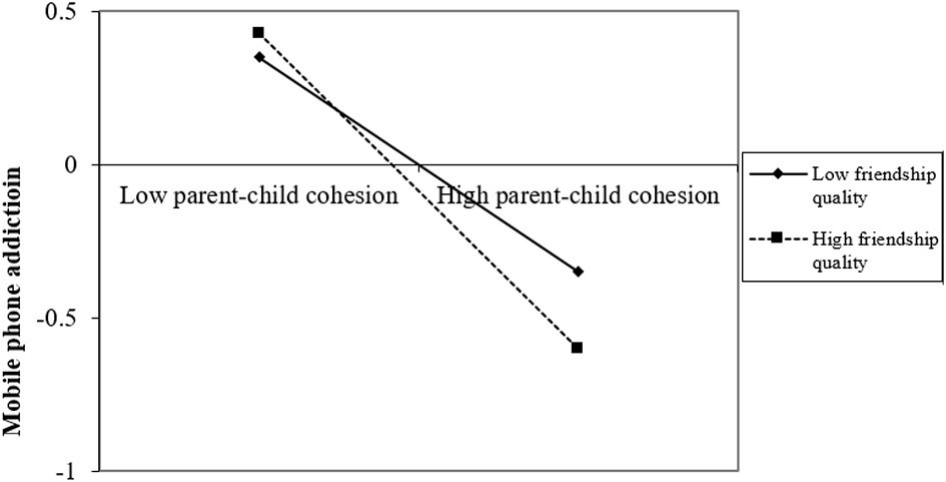
The integrated model.

Simple slope tests revealed that the effect of parent-child cohesion on mobile phone addiction was greater for adolescents with high friendship quality (*b* simple = −0.51, *t* = −12.13, *p* < 0.001) than for adolescents with low friendship quality (*b* simple = −0.35, *t* = −10.54, *p* < 0.001). Figure 3 illustrates the interaction plot.

## 5. Discussion

Based on the parental acceptance-rejection theory (PAR Theory) and the model of individual ↔ context relations, this study examined the potential mechanism underlying the link between parental phubbing and mobile phone addiction. It shed light on how parent-child cohesion and friendship quality play their roles on the said association. The results of the present study expanded the knowledge of the well-established relationship and may contribute to provide viable interventions in the future.

### 5.1. Parental phubbing and mobile phone addiction

The result indicated that parental phubbing was positively associated with mobile phone addiction, which demonstrated H1. Parenting practices are directly related to adolescent outcomes according to the integrative model of parenting (Durbin et al., 1993). In our findings, parental phubbing was a kind of negative parenting practice associated with mobile phone addiction for teenagers.

Consistent with previous studies, negative parenting practices are positively associated with mobile phone addiction, including harsh parenting, parenting by lying, neglect and rejection (Sun et al., 2019; Zhang et al., 2021; Wei et al., 2022a,b). For adolescents, parents are expected to provide much power and support. However, if parents failed to meet their needs, they might over-evaluate the severity of parents' failure and respond with a complaining style (Sanders and Becker-Lausen, 1995), which could lead to addictive behavior.

Different from explicitly negative parenting practice, parental phubbing has its own behavioral and emotional characteristics that result in less attention (Wei et al., 2021). First, explicitly negative parenting practices usually involve observable behaviors, such as hitting, kicking, scratching, that parents and the public are aware of. Therefore, parents are more likely to realize that harsh parenting is potentially hazardous and problematic. However, the behaviors of parental phubbing are minor, unnoticeable, and insidious (Xie and Xie, 2020; Zhang et al., 2021), sometimes parents are accustomed to phubbing as soon as they hear a beep on the phone or receive a message. It's more difficult for parents to discern the negative influence on their teenagers. Second, harsh parenting practices, such as rejection and aggression, are usually associated with intense negative emotions such as anger, which is easier for parents to be aware of and reflect on the harm of their harsh parenting. Previous study has proved that parents' emotional stability serves as a protective factor alleviating the impact of parental phubbing (Qu et al., 2022). Parental phubbing is usually carried out during a stable emotion of the parents rather than during a strongly negative emotion. Moreover, more often parental phubbing occurs because parents have to, rather than they are willing to, in consideration of working or social demands. Therefore, they pay little attention to the potential negative consequences.

In line with previous studies showing a significant association between parental phubbing and mobile phone addiction, parental phubbing has a wide and stable negative impact on their children in both behavioral and emotional influence (Xie et al., 2019; Niu et al., 2020; Zhang et al., 2021; Zhao et al., 2022). We consider it is important to raise awareness of the negative consequences of parental alienation. Furthermore, with the economic and social development and the popularity of mobile phones, the problem of mobile phone addiction among teenagers has become a major and urgent issue for families and school education. Based on the results of this study, if parents intend to reduce the risk of mobile phone addiction among teenagers, they can no longer ignore the seemingly harmless parental phubbing.

### 5.2. The mediation of parent-child cohesion

The result indicated that parental phubbing was positively associated with mobile phone addiction through the mediating role of parent-child cohesion, which demonstrated H2. This finding provides literature that problematic parenting practices are associated with mobile phone addiction through the mediating role of family factors. Consistent with prior studies, parental phubbing is a constant source of mobile phone addiction for adolescents and highlighted the mediating effect of family factors, such as parent-child relationship and attachment styles (Hong et al., 2019; Xie et al., 2019; Liu et al., 2021).

In the first stage of the mediation effect, the present study found that parental phubbing was negatively related to parent-child cohesion. As parental phubbing is equivalent to social exclusion (David and Roberts, 2017), adolescents who were phubbed by their parents would feel neglected and rejected (Borelli et al., 2015; Xie and Xie, 2020). The rejected reactions of significant others, particularly parents, have been shown to be a critically negative contributor to parent-child relationship (Terry, 2004; Matejevic et al., 2015) and have been postulated by parental acceptance-rejection theory (Rohner et al., 2005). Moreover, parent-child cohesion, a crucial indicator of the quality of parent-child relationship (Zhao et al., 2015), is similarly negatively affected.

For the second stage, we found that patent-child cohesion negatively associated with mobile phone addiction. This is in accordance with previous studies which have shown a positive correlation between terrible family environment and addictive behavior in teenagers (Ko et al., 2007; Salimi et al., 2016; Chung et al., 2019). Adolescents who experience lower family cohesion may be more likely to seek out external attention, including through mobile phones via the Internet (Sarour and El Keshky, 2022), as a means of emotional regulation (Park et al., 2008), which could lead to over-dependence on mobile phones.

Compared to the mediating factor assumed in the prior studies (Niu et al., 2020; Zhang et al., 2021; Wang et al., 2022; Wei et al., 2022a), the mediation of parent-child cohesion underlines the important role of environmental variable instead of personal variable. Therefore, effective prevention and intervention of mobile phone addiction should not only focus on individual factors, but also take into account environmental factors, especially the family environment. We encourage parents to develop good smartphone usage habits in order to create a harmonious and positive family environment. In addition, it is suggested that family therapy should be considered in the treatment of adolescents suffering from mobile phone addiction.

### 5.3. The moderation of friendship quality

Friendship quality played a moderating role in the association between parent-child cohesion and mobile phone addiction, which demonstrated H3. The results showed that parent-child cohesion had less effect on mobile phone addiction among adolescents with low friendship quality, whereas it had more effect on mobile phone addiction among adolescents with high friendship quality. In other words, friendship quality works to strengthen the negative effect of parent-child cohesion on mobile phone addiction.

Given the selection effect of friendship, adolescents are more likely to have close relationships with others who are like them, and are therefore more likely to share similar information, adopt similar attitudes and engage in similar behaviors (Lazarsfeld and Merton, 1954; McPherson et al., 2001). In Chang (2022)'s study, the selection effect is a primary mechanism for determining whether adolescents will engage in negative behavior, which implies that inappropriate behavior would be reinforced in the addictive group because its members would seek approval in the similarity group.

Previous research demonstrates that attitudes toward future addictive behavior are reinforced within a group of friends, linking friendship to anticipated behavior (Jones et al., 2004; Hall and Valente, 2007; Giletta et al., 2021). Selecting mobile phone addicts as friends is a major risk factor for previous addictive behavior and future intentions to indulge in mobile phone use. In the future, educators will be able to gather and discuss information about young people's everyday social experiences of friendship in order to develop a deeper understanding.

Friendship provides many different functions for children development, including warmth, affection, and intimacy (Bollmer et al., 2005). Adolescents with high friendship quality are more capable to feel warmth, love and intimacy from parents and family, their perception of social support level is higher. According to the main effect model, social support always creates a positive effect on physical health and reduce or abstain from addictive behaviors (Fried and Tiegs, 1993; Schwarzer and Knoll, 2007; Thomas et al., 2011; Taş and Öztosun, 2018). Adolescents with higher perception of social support are better able to use the strengths of the family and peers to reduce the risk of addiction, which means that parent-child cohesion would be more effective in this process. Therefore, our result recommends that parents are expected to improve parent-child communication and enhance parent-child relationship for adolescents with high friendship quality. In addition, to reduce the risk of mobile phone addiction, educational practitioners should pay more attention to students with low social support and provide psycho-educational services such as social skills development and communication skills. Consistent with previous studies, family environment and friendship quality have synergistic effects (Klauda and Wigfield, 2012; Kretschmer et al., 2016), especially in leading to externalization problems. Some researchers have found that both parents' and friends' addictive behaviors, such as smoking and drinking (Bauman et al., 2001; Hall and Valente, 2007; Lynch et al., 2015), may play a critical role in adolescent addiction. In contrast to drinking and smoking, mobile phone does not cause terrible damage to adolescents in a short period of time. However, many scholars have stated that mobile phone addiction can be more dangerous than other addictions, leading to sleep disturbance, anxiety, stress and fatigue (Goswami and Singh, 2016; Peraman and Parasuraman, 2016; Zhang et al., 2022).

Moreover, the current study enriched the framework of the moderators on the association between parental phubbing and mobile phone addiction. Individual factors, such as gender, refusal self-efficacy, and self-esteem, were examined as moderating variables to test the relationship between parental phubbing and mobile phone addiction (Xie et al., 2019; Zhang et al., 2022; Zhao et al., 2022). The current study examined friendship quality, a situational variable, as a moderator on the association, providing some insights for future research.

## 6. Limitations and implications

This study has several limitations that need to be taken into account. Firstly, because our research was a cross-sectional design, we cannot rule out the possibility of drawing casual conclusions. Experiments and longitudinal studies can be conducted in the future to establish the directions between variables. Secondly, the data in this study were homologous. Response bias due to self-reporting could cause shared variance and exaggerate the results of the study. More broader and representative samples, such as collecting data from parents and peers, are required in further research. Thirdly, we merely studied adolescents from mainland China, so generalization to populations in other cultures should be made with caution. Future research can replicate the findings in adolescents from other locations.

This study has both several theoretical and practical implications. From a theoretical perspective, the results of this research enrich the study of the mechanism of parental phubbing on mobile phone addiction among adolescents, in which friendship quality has been creatively considered as part of this process. Friendship quality makes the role of parent-child cohesion more comprehensive and effective. From a practical point of view, parents should “put down” their mobile phones when accompanying their children. It is crucial to urge parents to be sensitive to adolescents. In addition, adolescents are expected to improve the quality of their friendships, which is helpful in enhancing the advantage of a good family environment. Moreover, the authority is hoped to attach great importance to young people's addiction to smartphones and issue guidelines to remind parents of their educational responsibilities.

## 7. Conclusion

In summary, the present study examined the relationship between parental phubbing and mobile phone addiction among Chinese junior high school students, as well as the mediating role of parent-child cohesion and the moderating role of friendship quality in this relationship. Our findings suggest that parental phubbing was not only directly associated with mobile phone addiction, but also indirectly through the mediating role of parent-child cohesion and the moderating role of friendship quality.

## Data availability statement

The raw data supporting the conclusions of this article will be made available by the authors, without undue reservation.

## Ethics statement

The studies involving human participants were reviewed and approved by Qingdao University's Research Ethics Committee. Written informed consent to participate in this study was provided by the participants' legal guardian/next of kin.

## Author contributions

ZM conceived and designed the survey, performed the survey, and contributed to materials and analysis tools. ZM and WC analyzed the data. ZM, WC, WD, MW, and XF drafted the manuscript and contributed to literature research. ZM, WC, and WD revised the manuscript. All authors contributed to the article and approved the submitted version.
